# Assessment of operator performance during oocyte retrievals: residents’ learning curve and continuous monitoring of senior physicians

**DOI:** 10.1186/s12909-021-02615-w

**Published:** 2021-04-06

**Authors:** C. Verhaeghe, H. El Hachem, L. Inchboard, R. Corroenne, C. Dreux, P. Jeanneteau, G. Legendre, P. Descamps, P. Saulnier, P. May-Panloup, P. E. Bouet

**Affiliations:** 1 Department of Reproductive Medicine, Angers University Hospital, 49000 Angers, France; 2 Department of Reproductive Medicine, Clemenceau Medical Center, Beirut, Lebanon; 3 Clinical Research Center, Angers University Hospital, Angers, France

**Keywords:** Oocyte retrieval, Learning curve, LC CUSUM test, CUSUM test, Performance

## Abstract

**Background:**

The learning curve cumulative summation test (LC CUSUM test) allows to define an individualized learning curve and determine the moment when clinical proficiency is attained. After acquisition of the skills, the cumulative summation test (CUSUM test) allows to monitor the maintenance of the required level over time. The LC CUSUM test has been frequently used in the field of Obstetrics and Gynecology (Ob/Gyn) for several procedures, but only once for OR.

**Methods:**

We performed a retrospective study at Angers university hospital between May 2017 and September 2018. Seven Ob/Gyn residents and 5 senior physicians were included, and all OR performed during that time (*n* = 690) were analyzed. The performance index assessed was the oocyte retrieval rate (ORR), defined as the ratio of oocytes retrieved to follicles aspirated. We used the LC CUSUM test to analyze the learning curves of residents, and the CUSUM test to monitor the performance of senior physicians. An ORR ≥50% in 60% of retrievals was defined as the threshold for clinical proficiency.

**Results:**

Six hundred seventy-four oocyte retrieval (OR) were included: 315 were performed by residents, 220 by senior physicians, and 139 by both residents and physicians (mixed retrievals). Four residents (57%) reached the threshold after aspirating 82, 67, 53 and 46 ovaries, respectively. The mean number of ovaries aspirated in order to reach clinical proficiency was 62, and the mean number of weeks needed was 21. The duration of the learning period varied between 26 and 80 days. Two senior physicians (40%) remained proficient across the duration of the study, while two physicians (40%) had one statistically “suboptimal” OR, and one physician (20%) had two suboptimal retrievals.

**Conclusion:**

There is a large variability in the duration of the learning period and the number of procedures needed for a resident to master OR. Senior physicians maintain an adequate performance.

## Background

In France, approximately 6000 oocyte retrievals (OR) are performed each year, in 101 In Vitro Fertilization (IVF) centers [1]. OR is an invasive procedure that has a direct impact on the success rate of an IVF cycle. Indeed, several studies have now shown that, in the era of vitrification, the cumulative live birth rate is directly correlated to the number of oocytes retrieved [2]. In other words, the higher the number of oocytes retrieved, the higher the chances of success. It is therefore essential for physicians to master the procedure before performing it on a regular basis.

The training programs of Obstetrics and Gynecology (Ob/Gyn) residents and Reproductive Endocrinology and Infertility (REI) fellows differ between countries and continents, but some aspects can be somewhat similar. For instance, OR are usually considered straightforward procedures, and residents and fellows start their OR training by observing their teachers, before performing them assisted and finally performing them alone. However, very few studies have analyzed the learning curve of residents, or the number of retrievals needed to attain clinical proficiency, and the results have been quite heterogenous [3, 4]. Moreover, according to the latest European Society of Human Reproduction and Embryology (ESHRE) report [5], there are currently no recommendations by national fertility societies across Europe concerning the minimal number of OR required for a physician to become a certified specialist.

Nowadays, establishing a learning curve is crucial to maintain quality assurance and patient safety. The learning curve cumulative summation test (LC CUSUM test) was specifically designed to monitor the performance levels in the learning period of a procedure [6, 7]. Once the score reaches a predefined limit, the performance level is deemed adequate [7]. The maintenance of the performance level afterwards can be monitored using the CUSUM test. These tests have been used in several medical and surgical fields [7–13], as well as in the field of Obstetrics and Gynecology, for many procedures such as fetoscopic laser surgery [14], ultrasound diagnosis of endometriosis [15, 16] and hysteroscopy [17]. In the field of REI, it has been used for embryo transfer and 3-dimensional sonographic monitoring during ovarian stimulation, but only once for it has OR [18–20]. Therefore, we aimed to analyze the learning curves of residents training to perform OR using the LC CUSUM test, and to control the performances of senior physicians using the CUSUM test.

## Methods

We undertook a retrospective unicentric study at the REI department of the Angers university hospital, where approximately 550 OR are performed per year. We included all OR performed by residents and senior physicians between May 2017 and September 2018.

The residents group included all residents rotating in the REI department. They came from 3 different specialties: Ob/Gyn, medical gynecology, and endocrinology. The rotation lasts 6 months (1 semester), with only a few exceptions where it lasts 3 months (residents can choose to have 3 months in the REI and 3 months in the fetal ultrasound department). Our department has 2 or 3 residents per semester, most of them at the end of their training (4th and 5th year residents in Ob/gyn).

The seniors group included all physicians performing OR at our center: 3 senior physicians specialized in REI, and 2 fellows (senior B and E) with a significant experience in OR.

All the participants signed an informed consent before being included in the study.

We excluded from the study: (1) residents who had already performed OR prior to their rotation in our department; (2) OR with missing technical sheets; (3) OR with incomplete technical sheets (missing number of follicles or oocytes retrieved, missing information about the side aspirated or the identity of the operator).

Our main outcome measure was the oocyte retrieval rate (ORR), widely considered the best performance indicator. The ORR is defined as the number of oocytes retrieved divided by the number of follicles aspirated.

### The oocyte retrieval

All OR were performed in the operating theater, under light sedation, 36 h after the trigger injection. A transvaginal ultrasound was first performed to assess the ovaries and the feasibility of the retrieval. A 17 Gauge needle was then mounted on the probe, and inserted through the vagina to aspirate the follicles. The policy at our center is to aspirate all follicles with a mean diameter ≥ 8 mm. We do not aspirate follicles between 2 and 7 mm, since it is technically challenging with a very low chance of obtaining a mature oocyte [21]. The follicular fluid was recovered in numbered tubes, kept at 37 °C, and the operator counted the number of follicles in each tube. Once an ovary was fully aspirated, the needle was withdrawn and flushed with culture media (Life global, Guilford, USA) in order to completely empty the tubing (HOC® ART, CCD, France). New aspiration tubes were numbered and added, and the needle was inserted back to aspirate the second ovary. The name of the operator and the number of follicles aspirated were written on a technical sheet that was attached to each aspiration tube, and they were both transferred immediately to the laboratory, where the follicular fluid was analyzed, and the number of oocytes recovered per tube added to the technical sheet. At the end of the retrieval, the numbers were added and the ORR was calculated per ovary and per operator.

### The residents’ training

At the time of the study, the residents’ training proceeded as follows: During the first weeks, they observed senior physicians perform OR, and assisted them with straightforward tasks such as changing aspiration tubes (observation period). After that, they started aspirating one side in straightforward cases with easy access to the ovary, while the senior physicians aspirated the second (mixed retrievals) (learning period). And finally, when the performance level was considered satisfactory, residents were allowed to perform complete retrievals, in easy as well as more complicated cases, with the senior physician present and ready to intervene only in difficult cases, or when the resident was unable to proceed safely (autonomy period).

### Patients’ and IVF cycles’ characteristics

The demographic, social, and medical characteristics of the patients were retrospectively collected and compared according to the operator (resident, senior physician, or mixed). Furthermore, the characteristics of the IVF cycles (rank, stimulation protocol, total FSH dose, duration of stimulation) were also compared according to the operator. We calculated mean values and standard deviations for continuous variables and percentages for categorical variables. We compared continuous variables with Student’s t test and categorical variables with chi-square and Fisher’s exact test.

### Learning curves cumulative summation test (LC CUSUM test)

The residents’ performance levels were followed using the LC CUSUM test. This test was developed to identify when an intervention has reached an adequate, predefined level of performance. In a LC CUSUM test, the null hypothesis is that “a performance is inadequate” and the alternative hypothesis is that “a performance is adequate.” Therefore, the learning curves in this study are binary variables (adequate or inadequate performance), and there are no measuring units. At each procedure (t), the LC CUSUM test was calculated as follows: St = min(0, St – 1 + Wt); W = log((1-p_0_)/(1 - p_0_ – *d*)) in case of success, and W = log (p_0_/ p_0_ + *d*) in case of failure; p_0_ = acceptable failure rate; *d* = acceptable deviance from acceptable performance to be detected. The successive procedures were plotted on the x-axis and the St on the y-axis [7]. The performance cannot be considered acceptable as long as the score remains in the continuation region (between the x-axis and the decision limit (h_LC_) [7]. The score increases with success and decreases with failure. A statistical threshold, *h. Lc*, is defined, above which the null hypothesis is rejected, and the performance considered adequate.

An ORR was considered adequate if ≥50%, and all retrievals with a lower rate were considered failures. The clinical performance was considered adequate if at least 60% of retrievals were successful with a standard deviation of 15%. The thresholds used were based on the mean oocyte retrieval rate of the senior physicians in our department for the years 2015–2016. Indeed, the main objective of the training is to allow residents to reach the performance level of senior physicians, thus allowing them to perform the procedure on their own. A *h. Lc* threshold of 6 was calculated so that the risk of declaring a resident competent when his performance was inadequate was 4.5% (false discovery rate or α error) and the probability of declaring a resident competent when his performance was indeed adequate was 100% (true discovery rate or power). The frequency with which each resident performed the procedures is shown in Fig. 2.

### Cumulative summation test (CUSUM test)

We used the CUSUM test to monitor the level of performance of the senior physicians. The CUSUM test was developed to quickly detect changes in the performance levels, so that necessary action is taken if needed. The null hypothesis assumes that the performance is adequate (the process is “in control”). If the hypothesis is rejected, the performance is considered inadequate (process is “out of control”). The CUSUM score increases with each inadequate performance or failure and decreases with each successful performance. When the score crosses the threshold *h.c.*, the null hypothesis is rejected and, as long as the score remains below the threshold, the performance is considered adequate. We used the same criteria to consider an ORR and clinical performance adequate (≥50 and 60%, respectively). The *h.c* threshold was as fixed at 1.2 so that the probability of declaring a performance inadequate when it was adequate (false discovery rate or α error) was 1.7%, and the probability of declaring a performance inadequate when it was adequate (true discovery rate or power) was 100%.

### Ethical approval

Our study was approved by the local committee on April 28, 2018 (n°2018/37).

## Results

**Fig. 1:**
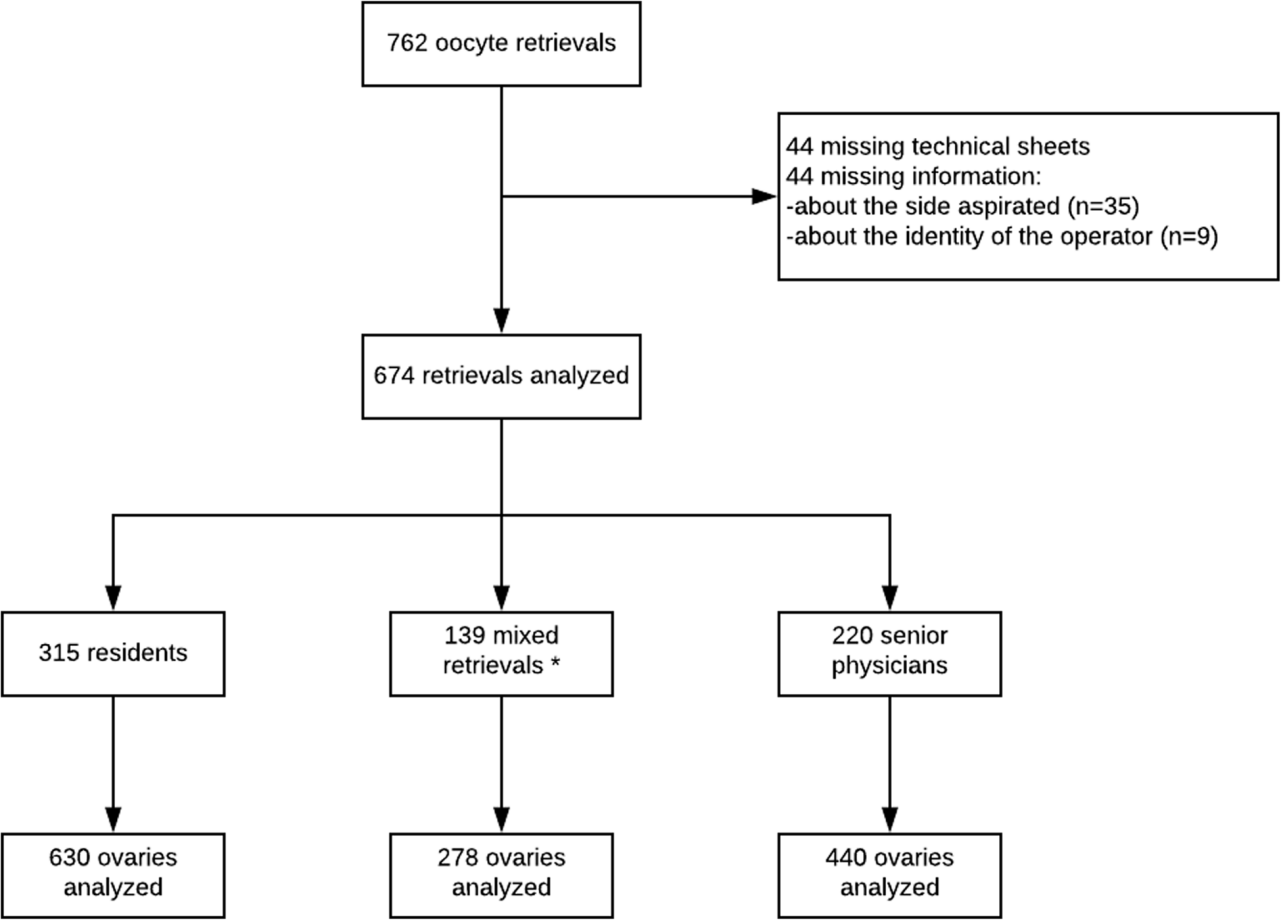
Flow chart. *Mixed retrievals: one side by the resident and one side by the senior physician

Across the duration of the study, 762 OR were performed: 44 were missing the technical sheet, while another 44 had missing information in the technical sheet. The total number of oocytes retrieved per ovary was missing in 35 cases, and the identity of the operator was missing in 9. In total, 674 OR were included: 315 were performed by residents, 220 by senior physicians, and 139 were mixed (one side by a resident and the other by a senior physician) (Fig. 1).

The seven residents (6 Ob/Gyn and 1 medical gynecology) who were rotating in the department across the duration of the study were included. Resident 5 was in the 3rd year of training but was only present for 2 months out of 6 because of maternity leave. Resident 6 was a 4th year medical gynecology resident who only spent 3 months in the REI department (partial time). Resident 1 was in the 4th year of training and had multiple consecutive absences in the last 2 months of training (wedding, yearly vacation, and sick leave). The remaining five had a full 6 months rotation in the department: 2 were in their fourth year and 3 were in their fifth year of training. The mean age of residents was 28 years.

### Patients and cycles characteristics

The characteristics of the patients and the IVF cycles are shown in Tables 1 and 2 (grouped based on the operator (residents, senior physicians or mixed). The patients in the “mixed group” were significantly younger (*p* = 0.005), had a significantly higher antral follicle count (AFC) (*p* = 0.04), and received a significantly lower dose of gonadotropins (*p* = 0.02) than patients in the other two groups. There were significantly more cases of male factor infertility (Oligoasthenoteratospermia) in the residents group compared to the senior physicians group (58.1% vs 41.3%, *p* = 0.001).

**Table 1:** Patients’ characteristics, grouped based on the operator performing the oocyte retrieval

	Operator			*P* value
	Resident	Mixed^a^	Senior physician	
n	315	139	220	
Age (years)	33.5 +/− 4.64	32.5 +/− 4.8	34.1 +/− 4.4	0.005
Body Mass Index (kg/m^2^)	23.9 +/− 5.45	24.2+/− 5.3	24.1+/− 5.3	0.90
Smoking status				0.58
Non-smoker	187 (66.8)	77 (66.4)	130 (67.4)	
Smoker	59 (21.1)	26 (22.4)	48 (24.9)	
Former smoker	34 (12.1)	13 (11.2)	15 (7.8)	
Baseline E2 (pg/mL)	44.4 +/− 93.3	37.5 +/− 24.3	40.1 +/− 35.0	0.43
Baseline FSH (UI/L)	7.4 +/− 3.5	6.8 +/− 3.1	7.6 +/− 4.1	0.08
Baseline LH (UI/L)	6.4 +/− 17.9	5.3 +/− 3.5	5.4 +/− 3.2	0.62
Baseline AMH (ng/mL)	2.78 +/− 3.07	3.4 +/− 4.4	2.9 +/− 3.5	0.34
Antral Follicle Count	18.6 +/− 11.1	21.3 +/− 12.2	17.9 +/− 11.3	0.04
Etiology of infertility^b^				
Endometriosis	44 (14.0)	24 (17.3)	27 (12.3)	0.41
Tubal factor	11 (4.1)	3 (2.6)	8 (4.1)	0.76
Oligoasthenoteratospermia	183 (58.1)	73 (52.5)	90 (41.3)	0.001
Unexplained	66 (20.9)	34 (46.8)	70 (31.8)	0.09
Ovulation disorders				0.46
• Ovarian insufficiency	40 (12.7)	12 (8.6)	34 (15.4)	
• PCOS^c^	45 (14.3)	21 (15.1)	30 (13.6)	

Data are expressed as n (percentage) or mean +/− standard deviation

^a^Retrieval performed during the learning period: one side is aspirated by the resident and the other side by the senior physician

^b^Some couples had several causes of infertility

^c^Polycystic Ovary Syndrome

**Table 2:** IVF cycles characteristics grouped based on the operator performing the oocyte retrieval

	Operator			*P* value
	Resident	Mixed	Senior physician	
n	315	139	220	
Rank of IVF cycle				0.27
1	188 (59.7)	84 (60.4)	113 (51.4)	
2	71 (22.5)	25 (18.0)	58 (26.4)	
3	32 (10.2)	18 (12.9)	21 (9.5)	
≥ 4	24 (7.6)	12 (8.7)	28 (12.7)	
ICSI	173 (54.9)	72 (51.8)	102 (46.4)	0.15
Type of protocol				0.054
Agonist	25 (7.9)	13 (9.4)	21 (9.5)	
Antagonist	287 (91.1)	126 (90.6)	191 (86.8)	
Natural modified cycle	3 (1.0)	0 (0.0)	8 (3.6)	
Total dose of FSH per cycle (UI)	2519.7 (1166.9)	2215.7 (978.4)	2366.4 (1131.3)	0.02
Duration of stimulation	10 (2.44)	9.6 (1.5)	9.5 (2.0)	0.02
Serum Progesterone level on trigger day	0.73 (0.43)	0.8 (0.4)	0.8 (1.4)	0.66
Serum Estradiol level on trigger day E2	1587.6 (1022.7)	1737 (1139.4)	1543.1 (1045.1)	0.26
Trigger type				0.55
Recombinant hCG	276 (87.6)	114 (82)	186 (84.5)	
GnRh agonist	12 (3.8)	9 (6.5)	10 (4.5)	
Double trigger (rhCG + agonist)	27 (8.6)	16 (11.5)	24 (11)	

### Assessment of residents’ learning curve

**Fig. 2:**
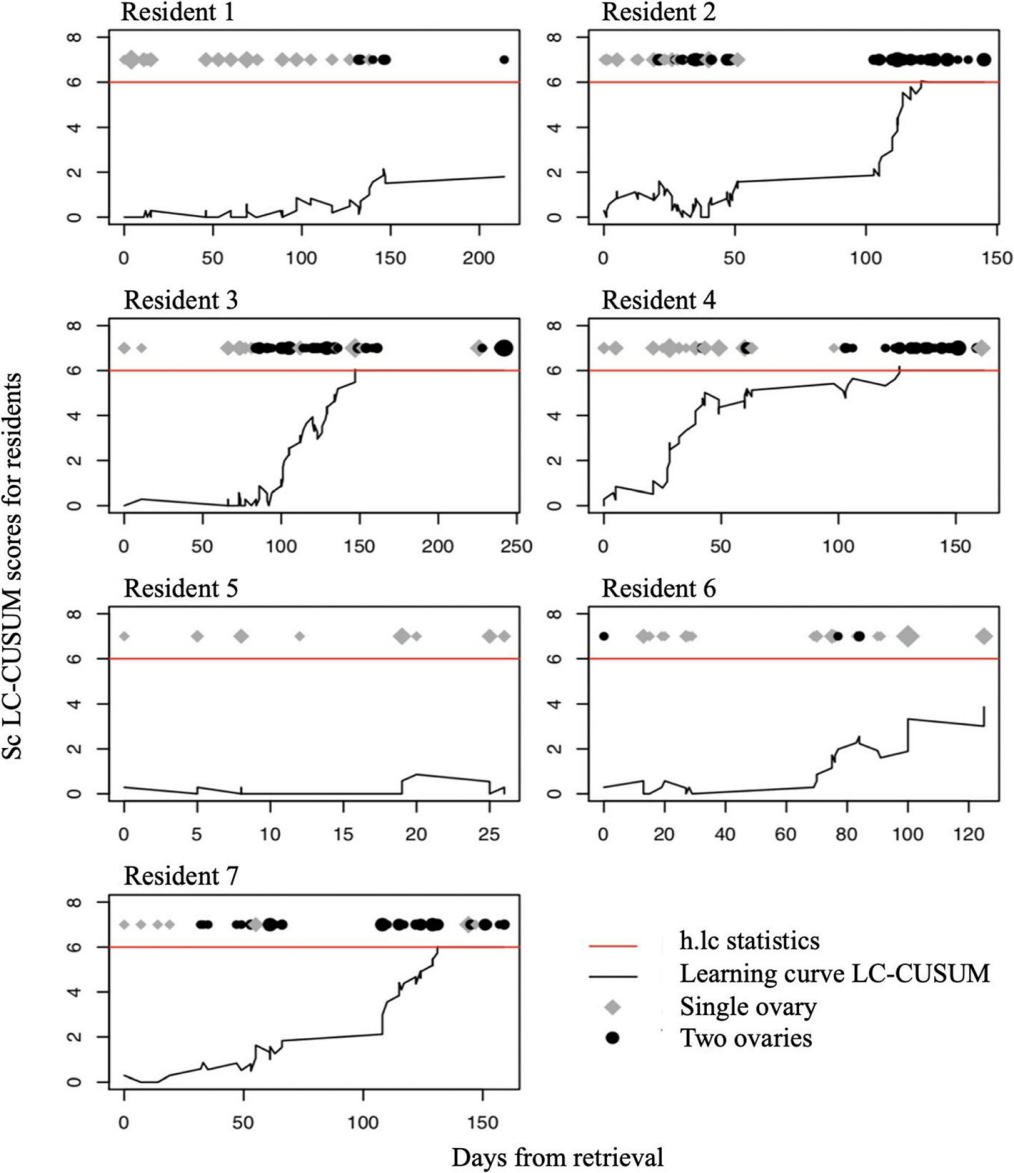
Learning curve cumulative summation test (LC CUSUM) graph for residents performing oocyte retrievals. The score is plotted on the y axis and time on the x-axis (number of days). The red line represents the threshold h.lc, above which the performance level is considered adequate. The prolonged periods without retrievals correspond to the periods where the department was closed (last week of December and 3 weeks in August), or when the residents were on vacation

The residents’ learning curves are shown in Fig. 2. Residents 2, 3 4 and 7 crossed the *h.lc* threshold respectively after the 82nd, 67th, 53rd, and 46th ovary. The mean number of ovaries aspirated before reaching clinical proficiency was 62, and the mean number of weeks needed was 21. Residents 1, 5 and 6 did not reach the threshold for clinical proficiency.

For residents 1, 2, 3, 4, and 7 (the residents who had a full 6 months rotation), the learning period was respectively 60, 26, 80, 32 and 47 days, respectively. The mean learning period was 49 ± 22 days. The median number of ovaries aspirated during that time was 21 [interquartile range (IQR); 18.5–22], with a minimum of 10 and a maximum of 27 ovaries.

For residents 2, 3, 4 and 7, the time interval between the beginning of the autonomy period and the attainment of an adequate performance level was 16, 32, 74 and 130 days, respectively. The mean duration was 63 ± 51 days.

Three residents did not reach the threshold during the observation period: two of them had only been training in the department for 2 et 4 months (maternity and multiple absences respectively), and one had a partial training schedule (50%).

### Assessment of performance of senior physicians

**Fig. 3:**
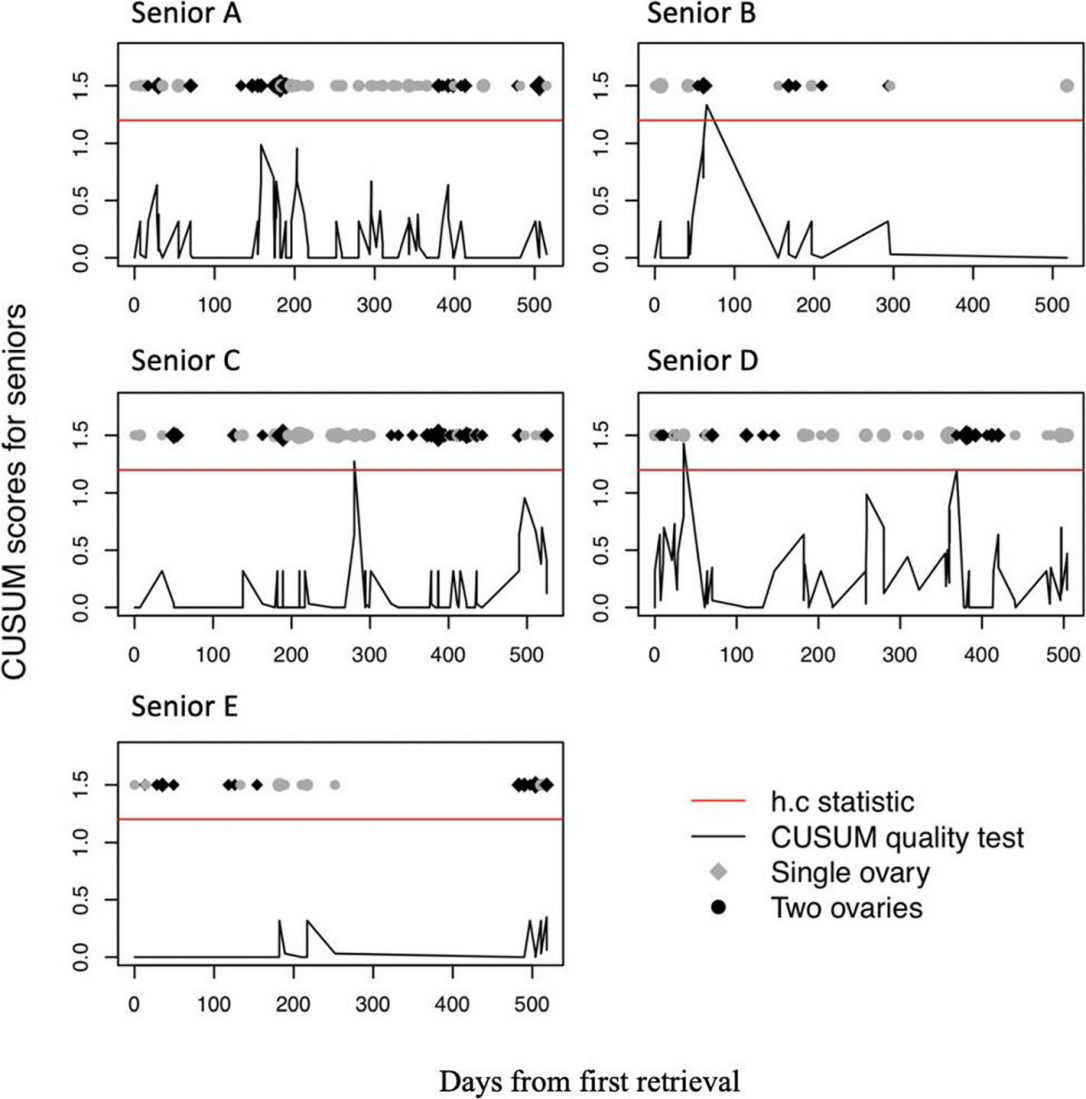
Cumulative summation test (CUSUM) graph for senior physicians performing oocyte retrievals. The red line represents the threshold h.c, above which the performance is considered inadequate. The score is plotted on the y-axis and time on the x-axis (number of days)

Figure 3 shows the performance level of senior physicians as assessed by the CUSUM test. Senior physicians A and E did not cross the threshold and thus always had an adequate performance across the duration of the study, while physicians B, C and D crossed the threshold once, once, and twice, respectively.

## Discussion

The main objective of our study was to analyze the residents’ learning curves while training to perform oocyte retrievals. We found a large variability in the residents’ performance levels, and we found that, in order to reach clinical proficiency, a resident must have a full rotation (at least 5 months of training) in the REI department, where he must regularly perform retrievals, and needs to aspirate a mean of 62 ovaries. We also found that the performance level of the senior physicians remained adequate across the duration of the study.

To the best of our knowledge, only two studies analyzed learning curves for assessing residents’ performances in OR [3, 4], using different methodologies and reporting somewhat different results. In a retrospective series, Goldman et al. [3] assessed the performance levels of REI fellows using a proficiency score. Out of the 6 fellows included, 4 of achieved the adequate level after 20 aspirations, one after 50, and one did not achieve it. The authors concluded that OR is rapidly mastered by fellows, with the majority reaching clinical proficiency after 20 procedures, and that there were no differences in the learning curves between them. Dessolle et al. [4], in a prospective trial performed over 6 months, analyzed the learning curves of 3 first year OB/Gyn residents with no previous exposure to OR, using the LC CUSUM test. They found a significant variability in the number of ovaries needed by each resident to reach the sufficient level: one needed 17, one needed 43, while the third did not reach the level even after 50 OR. The authors concluded that there is a large variability in the number of OR needed by different residents in order to reach clinical proficiency. In our study, the duration of the learning period was highly variable, going from 26 to 80 days, which is in accordance with Dessolle et al., while the median number of ovaries needed to puncture was 21, a relatively low number, that is in accordance with Goldman et al. Our results suggest that oocyte retrieval is a simple and straightforward procedure, which can be grasped with a low number of trials. The duration of the learning period, however, can be variable since it depends not only on the difficulty of the procedure, but on other external factors, such as the availability of the resident, the length of his rotation, and the activity of the department. Training should therefore be tailored according to each resident, and several international societies have included that notion in their recent recommendations. Indeed, in their latest practice guidelines, the American Society of Reproductive Medicine (ASRM) removed the minimum number of OR needed to be performed by residents and fellows during their training, and instead suggested that “physicians performing oocyte retrievals should have performed an adequate number of aspirations under direct supervision to demonstrate proficiency within a practice that meets these standards” [22]. In their guidelines [5], the ESHRE kept a minimum number of 30 OR to be performed under supervision to reach clinical proficiency but added that “this can vary depending on the type of training, background and progress of trainees”.

The patients in the mixed retrieval group were significantly younger and had a significantly higher AFC compared to the two groups, and there were significantly more cases of male factor infertility in the residents group compared to the seniors group. These findings are explained by the fact that, during the learning period, residents are progressively handed the straightforward cases, observing on one side and aspirating the other. In these situations, senior physicians will choose the accessible and good prognosis cases for their residents, hence the younger patients with a higher ovarian reserve.

We also assessed the residents’ performance during the autonomy period, where their level was considered satisfactory for them to perform the full retrieval (2 ovaries). Once again, we noted a large variability in the number of ovaries required (46 to 82) to reach the adequate performance level, as well as the time needed (16 to 130 days). Moreover, only the residents who completed their 6 months rotation in the department without notable absences reached the adequate performance level. The numbers needed in our study are in accordance to the ESHRE guidelines which state that at least 50 OR should be independently performed to obtain qualification [5].

The large variability in the performance levels between residents in a procedure considered “straightforward”, and the relatively high number of retrievals needed by some, constitute solid arguments for improving the current training programs. One of the solutions could be simulation training, which is currently used in different fields of medicine, surgery, and Ob/Gyn, and is recommended by most international Ob/Gyn societies. Recently, studies have reported on the use of simulation training for OR. In a prospective pilot study including 44 clinicians, Soave et al. [23] noted a significant improvement in efficiency, speed and accuracy in the total sample. The authors concluded that simulation-based training could be useful in the initial part of training, allowing residents and fellows to reach a predefined level of performance before operating on real patients. We recently performed an observational study [24] during a simulation workshop that included 46 residents from 23 different university hospitals. All residents found the training beneficial, and 87% were in favor of introducing it on the training program. These early studies are encouraging, but further studies are needed to assess the real impact of simulation training on the residents’ learning curve.

Our secondary objective was to analyze the performance levels of senior physicians. The CUSUM test showed that all senior physicians, attendings and fellows, maintained an acceptable level of performance across the duration of the study. Three physicians had suboptimal retrievals: 2 on one occasion and one on two occasions. Considering 220 retrievals were performed by senior physicians, these “accidents” could be explained by technical difficulties (adhesions, endometriosis, low follicle count …) rather than by a decrease in the overall performance level. These results are in accordance with the findings of Dessolle et al. [4] and suggest that, once clinical proficiency is achieved, the performance level remains stable over time, especially if the physicians keep performing the procedure regularly.

The main limitation of our study is the lack of external validity, since training programs, schedules and numbers of residents differ significantly between countries. Our findings are directly related to the French national training program, and could be useful to other centers in France, or other countries with training programs close to ours. However, these results are concordant with the few available literatures and the latest ESHRE and ASRM guidelines [5, 22].

## Conclusion

In conclusion, our study has shown that there is a large variability in the duration of the learning period and the number of procedures needed for a resident to master oocyte retrieval. Training must therefore be tailored to each resident, taking into consideration several factors such as the resident’s background, his previous experience, his availability, and the activity of the center. We found that an adequate performance level was reached after a mean number of 62 ovaries aspirated, and a full training schedule of 5 months during which OR are continuously preformed. On the other hand, senior physicians maintained an adequate level across the duration of the study, suggesting that the performance level remains adequate when the procedure is frequently performed.

## Data Availability

The datasets used and/or analysed during the current study are available from the corresponding author on reasonable request.

## Abbreviations

AFC: Antral Follicle Count
AMH: AntiMullerian Hormone
ASRM: American Society of Reproductive Medicine
CUSUM test: Cumulative summation test
E2: Estradiol
ESHRE: European Society of Human Reproduction and Embryology
FSH: Follicle Stimulating Hormone
IVF: In Vitro Fertilization
LC CUSUM test: Learning Curve Cumulative Summation Test
LH: Luteinizing Hormone
Ob/Gyn: Obstetrics and Gynecology
OR: Oocyte Retrieval
ORR: Oocyte Retrieval Rate
PCOS: Polycystic Ovary Syndrome
REI: Reproductive Endocrinology and Infertility
